# Methyl Gallate Enhances Post-Thaw Boar Sperm Quality by Alleviating Oxidative Stress and Preserving Mitochondrial Function

**DOI:** 10.3390/antiox14121465

**Published:** 2025-12-07

**Authors:** Yonghui Bu, Deming Shi, Jiahao Li, Xiaoxiang Jiang, Yuhan Chen, Zhenjun Wu, Wanxin Li, Li Li, Shouquan Zhang, Hengxi Wei

**Affiliations:** State Key Laboratory of Swine and Poultry Breeding Industry, National Engineering Research Center for Breeding Swine Industry, Guangdong Provincial Key Laboratory of Agro-Animal Genomics and Molecular Breeding, College of Animal Science, South China Agricultural University, Guangzhou 510642, China

**Keywords:** methyl gallate, boar semen, semen cryopreservation, antioxidant additive, sperm quality

## Abstract

Cryopreservation is a crucial technique for the long-term preservation of swine genetic resources. However, its efficiency remains limited by cryo-induced oxidative stress, which compromises sperm membrane integrity, mitochondrial function, and fertilizing capacity. Methyl gallate (MG), a naturally occurring polyphenolic antioxidant, has demonstrated strong free radical scavenging and lipid peroxidation inhibitory properties. This study aimed to evaluate the effects of MG supplementation on sperm quality and fertilization capacity during boar semen cryopreservation. Semen samples were cryopreserved in extenders containing different concentrations of MG (0, 10, 20, 30, and 50 µM). Post-thaw sperm quality, oxidative status, mitochondrial activity, apoptosis-related markers, and in vitro fertilization (IVF) outcomes were comprehensively assessed. The results showed that supplementation with 20 µM MG significantly improved post-thaw motility, viability, membrane and acrosome integrity, mitochondrial membrane potential, ATP content, and antioxidant capacity, while decreasing reactive oxygen species (ROS) and malondialdehyde (MDA) levels and reducing apoptosis (*p* < 0.05). Moreover, the expression of the anti-apoptotic protein BCL-2 was upregulated, whereas that of the pro-apoptotic protein BAX was downregulated. Sperm cryopreserved with 20 µM MG also exhibited a significantly higher IVF cleavage rate compared with the control group (*p* < 0.05). In conclusion, MG supplementation effectively enhanced boar sperm cryosurvival by maintaining membrane stability, improving mitochondrial function, and mitigating oxidative stress during freezing and thawing. These findings suggest that MG is a promising antioxidant additive for improving the efficiency of boar semen cryopreservation systems.

## 1. Introduction

The cryopreservation of boar semen is a pivotal technology for genetic resource exchange and long-term preservation in modern pig production. It enables the global dissemination of superior genetics, supports the conservation of rare or high-value boar lines, and plays a central role in biosecurity and pathogen-free germplasm exchange across regions [1,2,3]. Unlike cattle and sheep, intensive pig reproduction still largely relies on semen storage at 17 °C and conventional artificial insemination. However, liquid-stored semen is constrained by its short shelf life and limited transport distance and is highly vulnerable to sudden disease outbreaks or extreme weather. These limitations often result in reduced sperm motility, acrosomal damage, and plasma membrane disruption [4,5]. Therefore, developing reliable semen cryopreservation protocols is crucial for enhancing genetic progress, reducing disease transmission, and advancing precision breeding in pigs.

During the freeze–thaw process, boar spermatozoa are highly susceptible to cryoinjury, with oxidative stress being a major factor compromising motility, membrane integrity, mitochondrial activity, and chromatin stability, while also promoting apoptosis and impairing fertilizing capacity [6]. This vulnerability is largely attributed to the high content of polyunsaturated fatty acids (PUFAs) in the sperm plasma membrane [5]. Moreover, osmotic fluctuations and ice crystal formation during cryopreservation exacerbate ROS overproduction [7,8]. Excessive ROS accumulation triggers lipid peroxidation, DNA fragmentation, mitochondrial dysfunction, and apoptosis [9,10,11,12]. Therefore, mitigating oxidative stress is crucial for preserving sperm functionality during cryopreservation.

The supplementation of semen extenders with exogenous antioxidants is the primary strategy to counteract ROS and alleviate oxidative damage during cryopreservation [13,14]. Previous studies have demonstrated that natural compounds such as vitamins E and C, as well as plant polyphenols including resveratrol and quercetin, can scavenge free radicals, stabilize cell structures, and thereby improve sperm integrity to varying degrees [15,16,17,18]. In addition, antioxidant-enriched extenders have been associated with enhanced mitochondrial function and reduced apoptotic pathways [19,20]. Despite these advances, current antioxidant strategies remain limited: many additives show low bioavailability at cryogenic temperatures [21], and their protective effects often vary across semen batches and freezing protocols [22]. These shortcomings underscore the need for novel and more effective antioxidants that can consistently preserve sperm function while providing comprehensive protection against oxidative damage.

Methyl gallate (MG) is a naturally occurring polyphenolic compound widely distributed in plants and has attracted considerable attention due to its diverse biological activities [23]. MG has been reported to exhibit anti-tumor [24], antioxidant [25], anti-apoptotic [26], anti-inflammatory [27], antimicrobial [28], neuroprotective [29], and hepatoprotective effects [25]. In human cell studies, MG demonstrated strong ROS-scavenging capacity, preventing H_2_O_2_-induced oxidative stress, reducing lipid peroxidation, and enhancing cell viability [30]. In vivo, MG also displayed potent peroxynitrite (ONOO^−^) scavenging activity, significantly lowering ROS levels in animal models [31], and exerted hepatoprotective effects by modulating mitochondria-dependent apoptotic signaling pathways [32]. Although MG has been extensively investigated in medicine and biomedicine, its potential application in semen cryopreservation remains largely unexplored. Given its potent antioxidant and anti-apoptotic properties, MG may represent a promising candidate for protecting boar sperm against cryo-induced oxidative stress. Therefore, this study aimed to evaluate the effects of MG on boar sperm quality during cryopreservation and to preliminarily explore its underlying mechanisms.

## 2. Materials and Methods

Unless otherwise specified, all chemicals and reagents used in this study were purchased from Sigma-Aldrich (St. Louis, MO, USA). Methyl gallate (MG; HY-N2010) was obtained from MedChemExpress (Monmouth Junction, NJ, USA), and Detergent Equex STM (OEP; 13560) was purchased from Minitube (Munich, Germany). MG was dissolved in dimethyl sulfoxide (DMSO) to prepare a 10 mM stock solution and stored at −20 °C in the dark.

### 2.1. Preparation of Reagents and Freezing Extenders

The basic freezing extender (TCG) contained 22.4 g/L Tris, 11 g/L D-(+)-glucose, 14.8 g/L citric acid, and 35 g/L trehalose. Freezing extender I was prepared by supplementing the TCG extender with 20% (*v*/*v*) egg yolk, whereas freezing extender II was formulated by adding 6% (*v*/*v*) glycerol and 3% (*v*/*v*) Equex STM (OEP) to the TCG extender. The Modena thawing solution contained 27.5 g/L D-(+)-glucose, 5.65 g/L Tris, 6.9 g/L sodium citrate, 2.9 g/L citric acid, 1 g/L sodium bicarbonate, 2.35 g/L EDTA, 0.6 g/L penicillin, and 1 g/L streptomycin.

### 2.2. Semen Collection and Processing

Semen samples were collected from ten healthy, sexually mature Large White boars (*n* = 10) provided by Wens Foodstuff Group Co., Ltd. (Yunfu, China). Basic semen characteristics before processing were recorded, including ejaculate volume (210–280 mL), initial sperm concentration (2.5–3.2 × 10^8^/mL), and normal morphology rate (>80%). To minimize inter-ejaculate variation, ejaculates collected from different boars were pooled before analysis. Only pooled samples meeting these quality criteria were used. The semen was diluted with Modena diluent at a ratio of 1:1, then allowed to cool gradually to room temperature, and subsequently equilibrated in a cooling chamber at a rate of 0.2 °C/min until reaching 17 °C. The diluted semen was transported to the laboratory within 2 h for cryopreservation.

### 2.3. Sperm Cryopreservation and Thawing

The diluted semen was centrifuged at 850× *g* for 10 min at 17 °C to remove the supernatant extender. The sperm pellet was resuspended in freezing extender I to achieve a final concentration of 2 × 10^9^ sperm/mL. At this stage, the sperm samples were allocated into five treatment groups, each supplemented with a different concentration of MG (0, 10, 20, 30, or 50 μM). The suspension was then cooled to 4 °C at a controlled rate of 0.14 °C/min using a programmable cooling unit, and subsequently mixed with freezing extender II (1:1, *v*/*v*). The mixture was equilibrated at 4 °C for 30 min. The equilibrated semen was packed into 0.5 mL plastic straws (IMV Technologies, Laigle, France), sealed, and placed horizontally 4 cm above liquid nitrogen vapor for 15 min, followed by immersion in liquid nitrogen for storage. After seven days of storage, straws were thawed in a 50 °C water bath for 16 s and immediately diluted with pre-warmed (37 °C) Modena solution (1:5, *v*/*v*). The thawed sperm were incubated at 37 °C for 15 min prior to further assessments.

### 2.4. Assessment of Sperm Motility (CASA System)

An aliquot (8 μL) of diluted semen was loaded onto a pre-warmed (37 °C) disposable counting chamber (MAILANG, Nanning, China). Sperm motility parameters were analyzed using a computer-assisted sperm analysis (CASA) system (ML-300, version 5.6.0; MAILANG, Nanning, China) under standard settings. Detailed CASA settings were as follows: the acquisition rate was set at 60 frames/s, with 30 consecutive frames captured per field. The minimum and maximum sperm head area thresholds were set at 10 µm^2^ and 80 µm^2^, respectively, and the connectivity threshold was defined as 11 pixels. The minimum contrast was set at 80, and the minimum cell size threshold was set at 4 pixels to avoid false detection of debris. Sperm with an average path velocity (VAP) ≥ 10 µm/s were classified as motile, and progressive sperm were defined using a straightness (STR) threshold of ≥45%. The VAP and straight-line velocity (VSL) cut-off values were set at 20 µm/s and 15 µm/s, respectively. Five randomly selected microscopic fields, each containing at least 200 spermatozoa, were analyzed per sample. Although 200 sperm per frame were typically present, pilot tests confirmed no evidence of cell compression artifacts. Each experiment was repeated five times.

### 2.5. Thermo-Resistance Test (TRT)

After thawing, sperm samples were incubated at 37 °C for 4 h. Motility was recorded at 1 h intervals using the CASA system to evaluate the thermo-resistance of post-thaw sperm. All experiments were performed in quintuplicate.

### 2.6. Assessment of Sperm Plasma Membrane and Acrosome Integrity

Sperm plasma membrane integrity was evaluated using dual fluorescent staining with 6-carboxyfluorescein diacetate (6-CFDA) and propidium iodide (PI). Briefly, 100 µL of semen was incubated with 10 µL of 10 µM 6-CFDA and 10 µL of 12 µM PI at 37 °C for 10 min. Subsequently, 10 µL of the stained suspension was placed on a microscope slide and examined under a fluorescence inverted microscope (Olympus, Tokyo, Japan). At least 200 sperm per sample were counted. Plasma membrane integrity (%) was calculated as (number of intact sperm/total sperm counted) × 100.

Acrosome integrity was assessed using fluorescein isothiocyanate-labeled peanut agglutinin (FITC-PNA) in combination with Hoechst 33342 staining. A 50 µL semen sample was smeared on a slide and fixed with anhydrous methanol for 15 min. The sample area was covered with 50 µL of 100 µg/mL FITC-PNA solution and incubated in a humid chamber at 37 °C for 30 min, followed by three washes with PBS. Then, 50 µL of 1 µg/mL Hoechst 33342 solution was applied, followed by another three PBS washes and air-drying. The percentage of acrosome-intact sperm was determined under a fluorescence microscope by counting at least 200 sperm per sample, and calculated as (number of acrosome-intact sperm/total sperm counted) × 100.

### 2.7. Assessment of Mitochondrial Membrane Potential (MMP)

The mitochondrial membrane potential (MMP) of sperm was determined using the JC-1 assay kit (C2006, Beyotime Biotech, Shanghai, China). Briefly, thawed semen samples were treated with 10 µM carbonyl cyanide 3-chlorophenylhydrazone (CCCP) for 20 min as a positive control. Approximately 5 × 10^5^ sperm were incubated in 0.5 mL JC-1 working solution at 38 °C for 20 min. After incubation, samples were centrifuged at 700× *g* for 4 min at 4 °C, washed twice with JC-1 staining buffer (1×), and resuspended in buffer for analysis. The assessment of sperm MMP was performed using flow cytometry.

### 2.8. Determination of Sperm ATP Content

ATP levels were quantified using an ATP assay kit (S0026, Beyotime Biotech, Shanghai, China) according to the manufacturer’s protocol. The ATP working solution was freshly prepared, and ATP standard solutions (0.1, 1, and 10 µmol/L) were obtained by diluting a 1 mM ATP stock with 220 µL lysis buffer. For measurement, 200 µL of ATP working solution was added to each well of a 96-well plate and equilibrated at 26 °C for 5 min. Subsequently, 20 µL of sample or standard was added, and luminescence was measured using a microplate reader (Bio-Rad Model 680, Bio-Rad, Hercules, CA, USA). ATP concentration was determined from the standard curve and expressed as µmol/L.

### 2.9. Measurement of Reactive Oxygen Species (ROS) Levels

Intracellular ROS levels were determined using a ROS detection kit (Beyotime, Shanghai, China) based on DCFH-DA staining. Thawed semen samples were washed once with Modena thawing solution and centrifuged at 700× *g* for 3 min. The pellet was resuspended in DCFH-DA working solution and incubated at 37 °C for 15 min in a 5% CO_2_ incubator. After incubation, samples were washed twice with PBS and resuspended for measurement. The fluorescence intensity of DCF was analyzed using a flow cytometer (Beckman Coulter, Miami, FL, USA).

### 2.10. Determination of Total Antioxidant Capacity (T-AOC) and Malondialdehyde (MDA) Levels

Sperm total protein concentration, total antioxidant capacity (T-AOC), and malondialdehyde (MDA) content were measured using BCA (KGB2101, KeyGen Biotech, Nanjing, China), T-AOC (S0121, Beyotime Biotech, Shanghai, China), and MDA (S0131S, Beyotime Biotech, Shanghai, China) assay kits, respectively. Sperm samples were sonicated at 4 °C under a frequency of 19 kHz and a power of 295 W (5 s on/7 s off, eight cycles), followed by centrifugation at 12,000× *g* for 5 min. The supernatant was collected for further analysis.

For protein quantification, 20 µL of standard or sample was mixed with 200 µL of BCA working reagent and incubated at 37 °C for 30 min. Absorbance was measured at 562 nm using a microplate reader (BioTek Synergy H1, Winooski, Vermont, USA), and protein concentration was expressed as µg/µL.

For T-AOC measurement, the assay reagents and standard solutions (0.1–1.0 mM) were prepared according to the manufacturer’s instructions. The reaction mixture was incubated at 27 °C for 6 min, and absorbance was measured at 405 nm. T-AOC was expressed as mmol/g protein based on the standard curve.

For MDA analysis, 100 µL of sample or standard was mixed with 200 µL of MDA working solution and heated at 100 °C for 15 min. After cooling, samples were centrifuged at 1200× *g* for 8 min, and the absorbance of the supernatant was measured at 532 nm. MDA concentration was calculated using the standard curve (1–50 µmol/L) and expressed as µmol/mg protein.

### 2.11. Determination of Antioxidant Enzyme Activities

The activities of superoxide dismutase (SOD), catalase (CAT), and glutathione peroxidase (GPx) were determined using commercial assay kits (S0101S, S0051, S0056; Beyotime Biotech, Shanghai, China), following the manufacturer’s instructions.

For SOD activity, 20 µL of sperm lysate was mixed with 160 µL of WST-8/enzyme working solution and 20 µL of reaction initiation solution. The mixture was incubated at 37 °C for 30 min, and absorbance was measured at 450 nm. SOD activity was expressed as U/mg prot.

For CAT activity, 20 µL of sample was combined with 20 µL of CAT assay buffer and 10 µL of 250 mM H_2_O_2_ solution, then incubated at 25 °C for 5 min. Subsequently, 450 µL of termination solution was added, followed by dilution of 10 µL of the reaction mixture with 40 µL of CAT assay buffer. Then, 10 µL of this mixture was added to 200 µL of chromogenic working solution and incubated at 25 °C for 15 min. Absorbance was measured at 520 nm, and residual H_2_O_2_ was quantified using a standard curve. CAT activity was calculated according to the manufacturer’s formula and expressed as U/mg prot.

For GPx activity, 10 µL of sperm lysate was mixed with 173 µL of GPx assay buffer, 5 µL of 10 mM reduced glutathione (GSH), and 12 µL of 15 mM cumene hydroperoxide (Cum-OOH), followed by incubation at 27 °C for 20 min. After adding 6.6 µL of DTNB reagent, the reaction was continued for 10 min at room temperature. Absorbance was measured at 412 nm. GPx activity was calculated according to the manufacturer’s formula and expressed as U/mg prot.

### 2.12. Assessment of Sperm Apoptosis

Sperm apoptosis was evaluated using the Annexin V-FITC/PI apoptosis detection kit (C1062M, Beyotime Biotech, Shanghai, China). Thawed semen samples were centrifuged at 800× *g* for 7 min and washed twice with thawing solution. Approximately 3 × 10^5^ sperm were resuspended in 195 µL of binding buffer, then stained with 5 µL Annexin V-FITC and 10 µL PI for 30 min at room temperature in the dark. Fluorescence signals were analyzed using a flow cytometer (Beckman Coulter, Miami, FL, USA).

### 2.13. Reverse Transcription and Quantitative Real-Time PCR (RT-qPCR)

Total RNA was extracted from sperm using TRIzol reagent (Invitrogen, Carlsbad, CA, USA), and RNA concentration and purity were determined using a NanoDrop 2000 spectrophotometer (Thermo Fisher Scientific, Boston, MA, USA). Reverse transcription was performed using the PrimeScript RT Kit (RR036A, Takara, Tokyo, Japan). Gene-specific primers for BCL-2 and BAX were designed based on CDS sequences obtained from NCBI and synthesized by Sangon Biotech (Shanghai, China) (Table 1). Quantitative PCR was conducted using TB Green^®^ Premix Ex Taq™ II (RR820A, Takara, Tokyo, Japan) with GAPDH as the internal control. Reactions were performed on an Applied Biosystems 7900HT Real-Time PCR System under the following conditions: initial denaturation at 95 °C for 3 min, followed by 40 cycles of 95 °C for 10 s and 60 °C for 30 s. Melting curve analysis was then performed (95 °C for 15 s, 60 °C for 60 s, and 95 °C for 15 s). Each reaction was performed in triplicate, and relative gene expression levels were calculated using the 2^−ΔΔCt^ method.

### 2.14. Western Blot Analysis

Sperm protein samples were normalized to equal concentrations and mixed with 6× loading buffer (DL101, TransGen Biotech, Beijing, China) at a 5:1 ratio, then boiled at 100 °C for 10 min. Proteins were separated on 12% SDS-PAGE gels and transferred onto PVDF membranes (Millipore, Burlington, MA, USA). Membranes were blocked with 5% non-fat milk in TBST (20 mM Tris-HCl, 150 mM NaCl, 0.1% Tween-20; pH 8.0) for 1 h at room temperature, then incubated overnight at 4 °C with primary antibodies against BAX (1:1000, 2774S, CST, Danvers, MA, USA), BCL-2 (1:1000, 3498S, CST, Danvers, MA, USA), and β-Tubulin (1:1000, 2146S, CST, Danvers, MA, USA). After washing four times with TBST, membranes were incubated with HRP-conjugated goat anti-rabbit IgG (1:2000, 7074S, CST, Danvers, MA, USA) for 1 h at 27 °C. Protein bands were visualized using an ECL detection kit (6883S, CST, Danvers, MA, USA) and imaged with a Tanon imaging system (Tanon, Shanghai, China). Band intensities were quantified using ImageJ software (Version number: 1.53t, NIH, Bethesda, MD, USA), and β-Tubulin was used as the internal control. All experiments were repeated at least three times.

### 2.15. In Vitro Fertilization (IVF)

Porcine ovaries were obtained from a local slaughterhouse (Panyu Food Group Co., Guangzhou, China) and transported to the laboratory within 4 h at 37 °C. Follicular fluid was aspirated and allowed to settle at 37 °C for 15 min. The supernatant was discarded, and the remaining material was washed three times with 37 °C DPBS-PVA. Cumulus–oocyte complexes (COCs) were selected and cultured in 500 µL of in vitro maturation (IVM) medium (TCM-199 supplemented with 10 IU/mL equine chorionic gonadotropin, 10 IU/mL human chorionic gonadotropin, 0.1 mg/mL L-cysteine, 10% porcine follicular fluid, 10% fetal bovine serum, and 10 ng/mL epidermal growth factor) covered with mineral oil in a 4-well dish. The COCs were matured for 44–48 h at 38.5 °C in 5% CO_2_. Cumulus cells were removed using 0.1% hyaluronidase, and oocytes were washed and transferred to fertilization droplets. Thawed sperm were washed three times, resuspended in capacitation medium, and adjusted to a final concentration of 1 × 10^6^ sperm/mL. After capacitation for 30 min at 38.5 °C under 5% CO_2_, sperm were co-incubated with oocytes for 6 h. Presumptive zygotes were washed three times and cultured in PZM-3 medium at 38.5 °C, 5% CO_2_, and 100% humidity for 48 h. Cleavage rate (%) was calculated as (number of cleaved embryos/total number of matured oocytes) × 100%.

### 2.16. Statistical Analysis

All data were analyzed using GraphPad Prism 10 (GraphPad Software, San Diego, CA, USA). One-way analysis of variance (ANOVA) followed by Duncan’s multiple range test was used to assess differences among groups. All experiments were repeated five times, and data are presented as mean ± standard error (SEM). Differences were considered statistically significant at *p* < 0.05.

## 3. Results

### 3.1. MG Improved Post-Thaw Sperm Motility Parameters

As shown in Table 2, post-thaw sperm motility parameters were assessed, revealing that supplementation of the freezing extender with a specific concentration of MG significantly increased the percentages of total motile sperm (TM) and progressively motile sperm (PM), as well as straight-line velocity (VSL), curvilinear velocity (VCL), lateral head displacement amplitude (ALH), linearity (LIN), mean angular displacement (MAD), and straightness (STR) (*p* < 0.05). Specifically, the 20 μM MG group exhibited higher TM (82.59 ± 1.00% vs. 75.03 ± 1.86%) and PM (63.30 ± 0.44% vs. 53.93 ± 0.33%) compared to the control group, representing mean increases of 7.56% and 9.37%, respectively. In contrast, no significant differences were observed in average path velocity (VAP), wobble (WOB), or beat cross-frequency (BCF) between the MG-treated groups and the control group (*p* > 0.05). Furthermore, the 50 μM MG group showed significantly lower TM, VSL, VCL, VAP, BCF, and STR compared to the control group (*p* < 0.05).

### 3.2. MG Enhanced Post-Thaw Sperm Heat Tolerance

As shown in Table 3, post-thaw sperm were incubated at 37 °C for 1–4 h, and total motility was evaluated. Supplementation of the freezing extender with a specific concentration of MG significantly extended post-thaw sperm motility, with the 20 μM MG group maintaining significantly higher total motility and progressive motility across 1–4 h compared to other groups (*p* < 0.05) and retaining approximately 60% total motility after 4 h of incubation. However, the 50 μM MG group showed no significant differences in total motility and progressive motility at 1–4 h compared to the control group (*p* > 0.05).

### 3.3. MG Increased Post-Thaw Acrosome and Plasma Membrane Integrity

**Figure 1:**
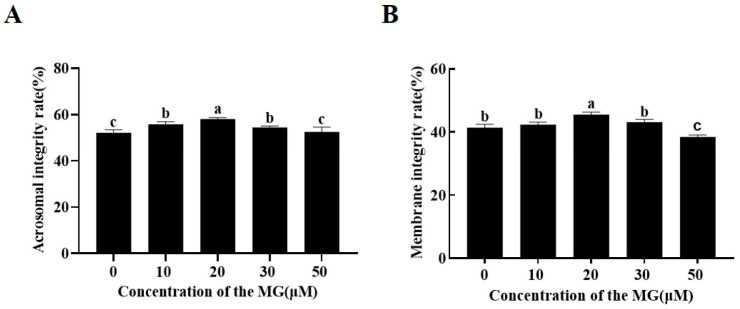
Effects of MG supplementation in the freezing extender on the acrosome and plasma membrane integrity of boar sperm after thawing. (**A**) Acrosome integrity. (**B**) Plasma membrane integrity. Values are expressed as mean ± SD (*n* = 5). Different letters indicate significant differences (*p* < 0.05).

Acrosome assessment using FITC-PNA/Hoechst 33342 double staining indicated that supplementation with 10, 20, and 30 μM MG significantly increased the percentage of sperm with intact acrosomes compared to the control group (*p* < 0.05), the 20 μM MG group showing the highest integrity. In contrast, the 50 μM MG group exhibited no significant difference from the control group (*p* > 0.05) (Figure 1A).

Evaluation of plasma membrane integrity using 6-CFDA/DAPI double staining revealed that the 20 μM MG group had a significantly higher percentage of intact sperm membranes compared to the control group (*p* < 0.05). However, the 50 μM MG group showed a significantly lower percentage compared to the control group (*p* < 0.05) (Figure 1B).

### 3.4. MG Elevated Post-Thaw Sperm MMP and ATP Levels

**Figure 2:**
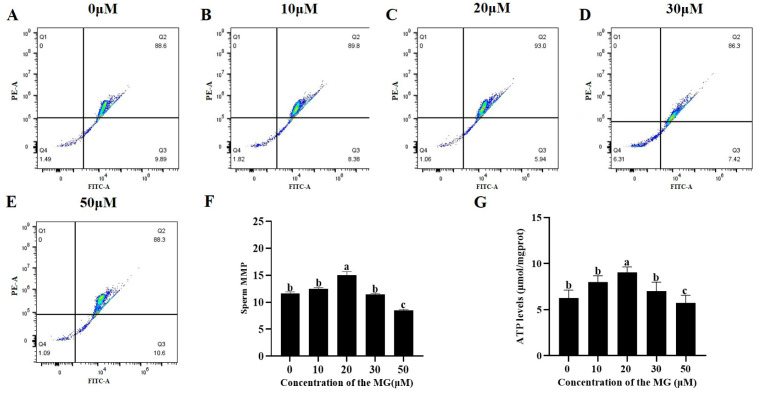
Effects of MG supplementation in the freezing extender on MMP and ATP content of boar sperm after thawing. (**A**–**E**) Flow cytometry evaluation of sperm MMP. (**F**) Sperm MMP. (**G**) Sperm ATP level. Values are expressed as mean ± SD (*n* = 5). Different letters indicate significant differences (*p* < 0.05).

The MMP and ATP levels of sperm from each group were determined following the freezing–thawing process. The results are shown in Figure 2, compared to the control group, supplementation with 20 μM MG in the freezing extender significantly increased both MMP and ATP levels (*p* < 0.05), whereas the 10 and 30 μM MG groups showed no significant differences (*p* > 0.05). In addition, the 50 μM MG group exhibited significantly lower MMP and ATP levels compared to the control group (*p* < 0.05).

### 3.5. MG Reduced Post-Thaw Sperm Oxidative Stress Levels

**Figure 3:**
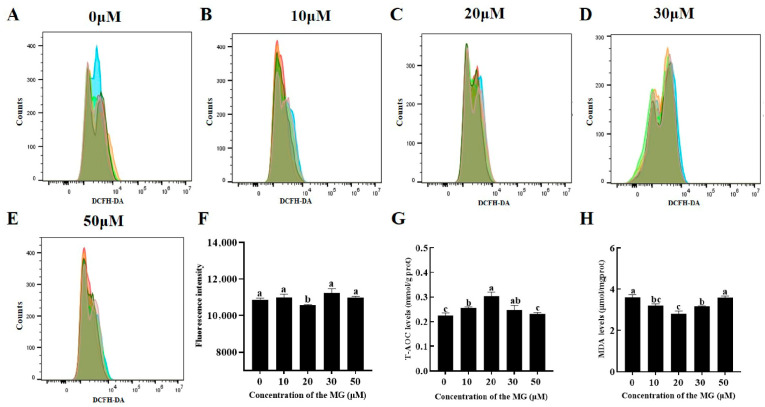
Effects of MG supplementation in the freezing extender on reactive oxygen species (ROS), total antioxidant capacity (T-AOC), and malondialdehyde (MDA) levels in boar sperm after thawing. (**A**–**E**) Flow cytometry evaluation of sperm ROS levels. Different colors represent biological replicate experiments. (**F**) Intracellular ROS fluorescence intensity of sperm analyzed using flow cytometry. (**G**) T-AOC levels. (**H**) MDA levels. Values are expressed as mean ± SD (*n* = 5). Different letters indicate significant differences (*p* < 0.05).

Levels of reactive oxygen species (ROS), total antioxidant capacity (T-AOC), and malondialdehyde (MDA) were quantified to evaluate oxidative stress. The 20 μM MG group showed significantly lower ROS levels compared to the control group (*p* < 0.05), while the 10, 30, and 50 μM MG groups exhibited no significant differences (*p* > 0.05) (Figure 3A–F). Furthermore, supplementation with 10, 20, and 30 μM MG significantly increased T-AOC levels and decreased MDA content compared to the control group (*p* < 0.05), with the 20 μM MG group displaying the highest T-AOC and lowest MDA. However, the 50 μM MG group showed no significant differences in T-AOC or MDA compared to the control group (*p* > 0.05) (Figure 3G,H).

### 3.6. MG Enhanced Post-Thaw Sperm Antioxidant Enzyme Activities

**Figure 4:**
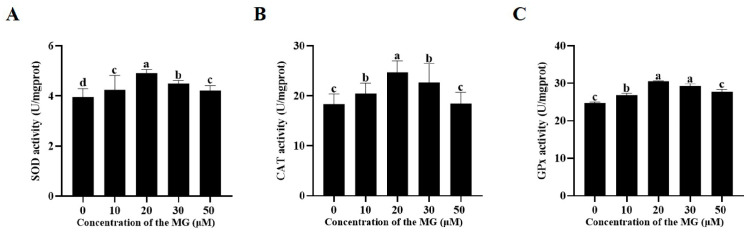
Effects of MG supplementation in the freezing extender on antioxidant enzyme activities in boar sperm after thawing. (**A**) SOD activity. (**B**) CAT activity. (**C**) GPx activity. Values are expressed as mean ± SD (*n* = 5). Different letters indicate significant differences (*p* < 0.05).

Activities of superoxide dismutase (SOD), catalase (CAT), and glutathione peroxidase (GPx) were assessed post-thaw. The results are shown in Figure 4, supplementation with MG in the freezing extender significantly increased SOD, CAT, and GPx activities compared to the control group (*p* < 0.05), with the 20 μM MG group exhibiting the highest levels across all enzymes. Specifically, all MG concentrations (10, 20, 30, and 50 μM) significantly elevated SOD activity (*p* < 0.05), though no significant difference was observed between the 50 μM and 10 μM MG groups (*p* > 0.05) (Figure 4A). For CAT, the 10, 20, and 30 μM MG groups showed significant increases (*p* < 0.05), whereas the 50 μM MG group did not differ from the control (*p* > 0.05) (Figure 4B). All MG concentrations (10, 20, 30, and 50 μM) significantly increased GPx activity (*p* < 0.05), with no significant differences between the 30 μM and 20 μM groups or between the 50 μM and 10 μM groups (*p* > 0.05) (Figure 4C).

### 3.7. MG Decreased Post-Thaw Sperm Apoptosis Levels

**Figure 5:**
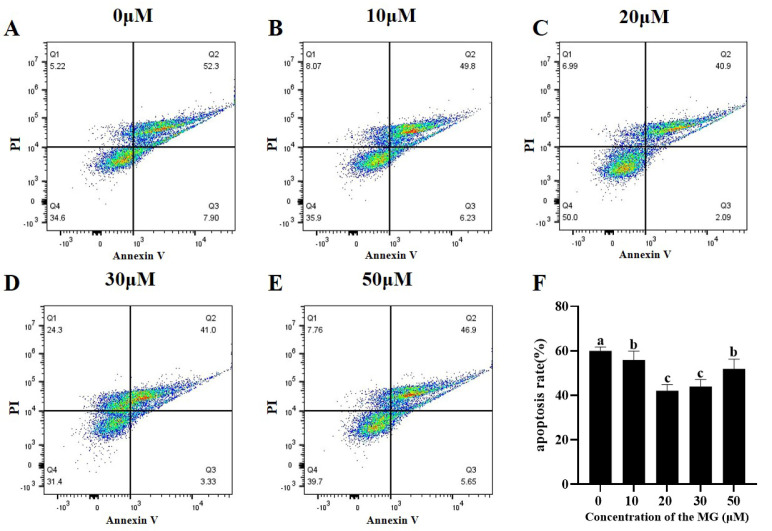
Effects of MG supplementation in the freezing extender on sperm apoptosis after thawing. (**A**–**E**) Flow cytometric analysis of apoptosis levels in thawed sperm treated with different MG concentrations. Different colored dots represent changes in cellular event density within the same sample. (**F**) Sperm apoptosis rate. Values are expressed as mean ± SD (*n* = 5). Different letters indicate significant differences (*p* < 0.05).

Apoptosis levels in post-thaw sperm were evaluated across groups. The results are shown in Figure 5, supplementation with MG in the freezing extender significantly reduced apoptosis compared to the control group (*p* < 0.05), with the 20 μM MG group showing the lowest levels.

### 3.8. MG Modulated Apoptosis-Related Gene and Protein Expression in Post-Thaw Sperm

**Figure 6:**
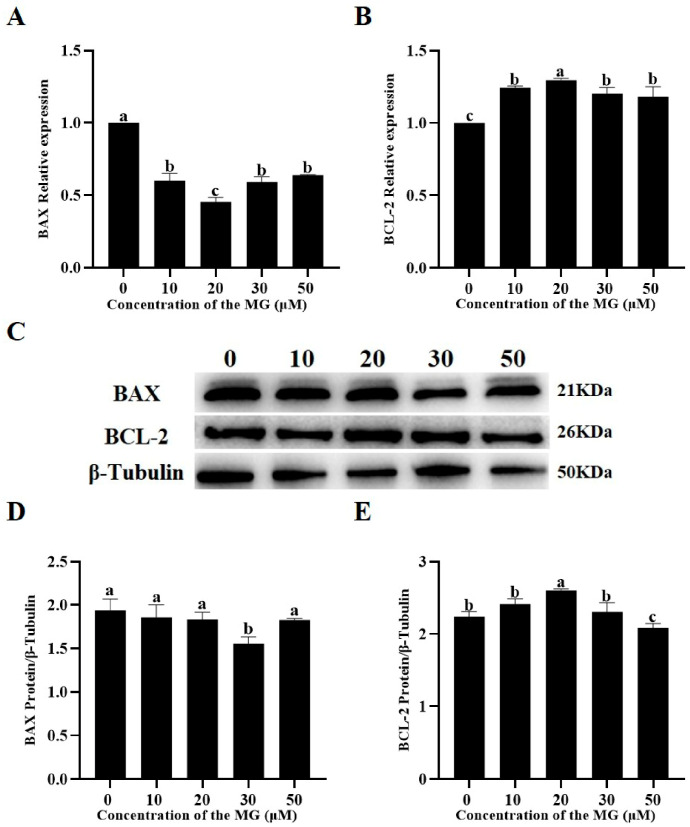
Effects of MG supplementation in the freezing extender on the expression of apoptosis-related genes and proteins in thawed boar sperm. (**A**) BAX mRNA expression level. (**B**) BCL-2 mRNA expression level. (**C**) Western blot analysis of BAX and BCL-2 protein expression in thawed sperm. (**D**) Quantification of BAX protein expression using ImageJ. (**E**) Quantification of BCL-2 protein expression using ImageJ. β-Tubulin was used as the internal control. Values are expressed as mean ± SD (*n* = 5). Different letters indicate significant differences (*p* < 0.05).

The effects of MG on apoptosis-related gene and protein expression were examined using RT-qPCR and Western blot. RT-qPCR results indicated that supplementation with 10, 20, 30, and 50 μM MG significantly upregulated mRNA expression of the anti-apoptotic factor BCL-2 compared to the control group (*p* < 0.05), with the 20 μM MG group showing significantly higher levels than the 10, 30, and 50 μM groups (*p* < 0.05) (Figure 6A). Conversely, all MG concentrations significantly downregulated mRNA expression of the pro-apoptotic protein BAX compared to the control group (*p* < 0.05), with the 20 μM MG group exhibiting significantly lower levels than the 10, 30, and 50 μM groups (*p* < 0.05) (Figure 6B). Western blot analysis revealed that 20 μM MG significantly increased BCL-2 protein expression compared to the control group (*p* < 0.05), whereas the 50 μM MG group showed significantly lower expression (*p* < 0.05) (Figure 6C,E). In addition, 30 μM MG significantly decreased BAX protein expression (*p* < 0.05), while the 10, 20, and 50 μM MG groups showed no significant differences from the control (*p* > 0.05) (Figure 6C,D).

### 3.9. MG Improved Post-Thaw Sperm Fertilization Capacity

Post-thaw sperm from groups supplemented with different MG concentrations were used for in vitro fertilization (IVF), and embryo cleavage was observed at 48 h. The results are shown in Table 4, supplementation with 20 μM MG in the freezing extender significantly increased the IVF embryo cleavage rate compared to the control group (*p* < 0.05), whereas the 50 μM MG group showed no significant difference (*p* > 0.05).

## 4. Discussion

During the freezing–thawing process, spermatozoa are exposed to drastic physicochemical stress, leading to excessive generation of reactive oxygen species (ROS). Because sperm cells possess limited endogenous antioxidant defenses, oxidative stress readily induces structural and functional damage, including lipid peroxidation, membrane disruption, and loss of motility [7,33]. Consequently, supplementation of exogenous antioxidants into cryopreservation media has been recognized as an effective strategy to preserve post-thaw sperm quality. Among potential candidates, plant-derived polyphenolic compounds have attracted particular attention for their potent antioxidative capacities, which can mitigate cryoinjury by scavenging ROS and stabilizing sperm membranes during freezing and thawing [34,35]. Methyl gallate (MG), a naturally occurring polyphenol, is well known for its antioxidant, anti-inflammatory, and antimicrobial properties [23]; however, its role in sperm cryopreservation and reproductive performance has not been explored. Given this context, the present study is the first to demonstrate that supplementation of 20 μM MG in the freezing extender significantly improved the post-thaw quality and fertilizing ability of boar sperm. Specifically, MG enhanced sperm motility, kinematic parameters, plasma membrane and acrosome integrity, mitochondrial membrane potential, ATP content, and antioxidant enzyme activities, while simultaneously reducing oxidative stress, lipid peroxidation, and apoptosis levels. These findings indicate that MG effectively alleviates cryo-induced damage and improves the functional competence of post-thaw spermatozoa.

Boar spermatozoa are particularly susceptible to cryodamage, primarily due to increased ROS production during freezing, which overwhelms the seminal plasma antioxidant defenses, disrupts membrane stability, and impairs mitochondrial function, ultimately leading to apoptosis [6,36]. Consistent with these observations, our results showed that untreated frozen–thawed sperm exhibited decreased motility and mitochondrial activity along with elevated apoptosis. Supplementation with 20 μM MG effectively counteracted these adverse effects, in line with reports that other polyphenols such as resveratrol (RES), silymarin (SMN), and epigallocatechin gallate (EGCG) exert protective effects during mammalian sperm cryopreservation [37,38,39]. However, excessive MG concentration (50 μM) failed to confer protection and even exhibited cytotoxicity, likely due to disruption of redox homeostasis leading to reductive stress [40]. This concentration-dependent pattern suggests that MG possesses an optimal protective window similar to other antioxidants, though with species- or context-specific differences.

Sperm motility is a direct indicator of cryopreservation efficiency and a key determinant of fertilization capacity [41]. High sperm motility is essential for successful transit through the female reproductive tract and fertilization of the oocyte [42]. Previous studies have demonstrated a positive correlation between sperm motility and in vitro fertilization (IVF) cleavage rates [43,44]. In our study, sperm treated with 20 μM MG exhibited significantly higher motility and kinematic parameters after thawing and maintained viability for a longer period during incubation, which corresponded to a higher IVF cleavage rate. The improvement in motility and fertilization competence may be associated with the protection of sperm plasma membrane and acrosomal integrity. The high proportion of polyunsaturated fatty acids (PUFAs) in boar sperm membranes renders them particularly vulnerable to ROS-induced lipid peroxidation [45,46]. MG supplementation (20 μM) effectively mitigated membrane damage and preserved acrosomal integrity, consistent with findings by Guo et al. [47], who reported that astaxanthin supplementation reduced lipid peroxidation and improved membrane stability during boar sperm cryopreservation. Malondialdehyde (MDA), a product of lipid peroxidation, is widely recognized as a biomarker of oxidative stress [48], while total antioxidant capacity (T-AOC) reflects the overall antioxidant status of spermatozoa [49]. Elevated ROS and MDA levels accompanied by decreased antioxidant capacity are key contributors to the poor quality of cryopreserved sperm [50,51]. The enzymatic antioxidant defense system—including superoxide dismutase (SOD), catalase (CAT), and glutathione peroxidase (GPx)—plays a critical role in scavenging ROS and maintaining redox balance [52]. In the present study, 20 μM MG markedly reduced ROS and MDA levels while enhancing T-AOC, SOD, CAT, and GPx activities, indicating that MG not only directly scavenges free radicals but also reinforces the endogenous antioxidant defense system. The superior effect of 20 µM MG may be attributed to achieving an optimal redox balance where ROS is effectively scavenged without inducing reductive stress. Similar results have been observed with other antioxidants, such as metformin, which reduced ROS and MDA levels and enhanced antioxidant enzyme activities in frozen–thawed sperm. Moreover, MG has been shown to alleviate oxidative stress and DNA damage by scavenging ROS and inhibiting lipid peroxidation in Madin–Darby canine kidney (MDCK) cells [53]. This dual antioxidative mechanism is particularly critical for sperm cryopreservation, where oxidative stress is a major cause of structural and functional impairment.

Mitochondria are the primary source of ROS in spermatozoa [54]. Excessive ROS can disrupt the mitochondrial membrane, leading to impaired mitochondrial function [55]. Since mitochondria are also responsible for ATP production via oxidative phosphorylation, maintenance of mitochondrial membrane potential (MMP) is essential for energy generation [56]. Dysregulation of the redox system results in loss of MMP, ATP depletion, and lipid peroxidation, ultimately reducing sperm motility [57,58]. In our study, 20 μM MG supplementation significantly increased MMP and ATP levels, suggesting that MG preserves mitochondrial functionality during cryopreservation and thus supports sperm motility and longevity. Because decreased MMP is also an indicator of early apoptosis [59,60], this mitochondrial protection may further contribute to MG’s anti-apoptotic effects. The mitochondrial apoptotic pathway is tightly regulated by BAX and BCL-2 proteins, which control mitochondrial membrane permeability [61]. A lower BAX/BCL-2 ratio indicates stronger anti-apoptotic capacity, and this ratio has been associated with sperm fertilization ability and embryo quality [62,63]. In the current study, MG supplementation (20 μM) significantly reduced sperm apoptosis and altered the expression of apoptotic markers by upregulating BCL-2 and downregulating BAX at both mRNA and protein levels. These findings suggest that MG enhances the anti-apoptotic potential of frozen–thawed sperm by modulating mitochondrial apoptotic signaling, consistent with previous studies showing that crocin reduced sperm apoptosis via the BAX/BCL-2 pathway [64]. Several studies have reported that high doses of MG can paradoxically induce oxidative imbalance and trigger apoptosis in somatic cells by promoting excessive ROS formation and impairing mitochondrial function [65]. Such duality aligns with our findings, in which only the highest MG concentration (50 μM) caused a decline in post-thaw sperm viability and mitochondrial activity. In contrast, the optimal dose (20 μM) effectively reduced apoptosis markers and maintained mitochondrial function, indicating that MG exerts a dose-dependent switch from protective antioxidant action to potential cytotoxicity when redox homeostasis is exceeded. This highlights the importance of carefully defining the effective yet safe concentration range of MG for application in boar semen cryopreservation. The increased cleavage rate in IVF further confirmed the functional relevance of this protection, as successful fertilization depends on intact motility, membrane integrity, and acrosomal function [66].

In the context of antioxidant-assisted cryopreservation, the protective effects of MG observed in this study are comparable to those reported for other natural antioxidants such as resveratrol, vitamin E, and quercetin. Resveratrol has been shown to enhance boar sperm motility and mitochondrial function by activating antioxidant enzymes and reducing lipid peroxidation [18], while vitamin E primarily stabilizes membrane lipids and prevents oxidative damage during freezing [67]. Quercetin, another plant-derived polyphenol, effectively improves post-thaw sperm viability and reduces ROS accumulation through its strong free radical-scavenging capacity [19]. Notably, the magnitude of improvement observed with 20 μM MG, particularly in motility, membrane integrity, MMP, and apoptosis markers were comparable to or in some cases greater than values reported for these standard antioxidants. This suggests that MG may achieve multi-level protection by simultaneously enhancing endogenous antioxidant defenses, preserving mitochondrial activity, and maintaining membrane stability, thereby offering a broader mechanistic spectrum than some traditional additives. Collectively, these comparisons underscore the potential of MG as a competitive alternative antioxidant for boar sperm cryopreservation.

This study provides new insights into the potential application of MG as a natural antioxidant additive in boar sperm cryopreservation. However, further studies across different species and in vivo assessment are warranted to verify whether its protective effects are universal and to elucidate the precise molecular mechanisms involved.

## 5. Conclusions

In conclusion, the present study demonstrated that supplementation with 20 μM MG significantly alleviates oxidative stress during boar sperm cryopreservation. MG effectively preserved sperm plasma membrane integrity, maintained mitochondrial function, modulated apoptotic signaling, and enhanced the overall antioxidant defense system, thereby improving post-thaw sperm quality and fertilization capacity.

## Figures and Tables

**Table 1:** Primer sequence.

Gene Name	Accession Number	Primer Sequence (5′-3′)	
		Forward Primer	Reverse Primer
BCL-2	XM_003121700.3	GGCAACCCATCCTGGCACCT	AACTCATCGCCCGCCTCCCT
BAX	XM_003355975.2	GCCGAAATGTTTGCTGACGG	CGAAGGAAGTCCAGCGTCCA
GAPDH	NM_001206359.1	CACGATGGTGAAGGTCGGAG	TTGACTGTGCCGTGGAACTT

**Table 2:** Effects of different concentrations of MG in the freezing extender on post-thaw sperm motility parameters of boars.

Parameters	0 μM	10 μM	20 μM	30 μM	50 μM
TM (%)	75.03 ± 1.86 ^c^	81.57 ± 0.83 ^a^	82.59 ± 1.00 ^a^	78.11 ± 0.87 ^b^	73.01 ± 0.35 ^d^
PM (%)	53.93 ± 0.33 ^d^	57.26 ± 0.52 ^b^	63.30 ± 0.44 ^a^	55.19 ± 0.54 ^c^	52.57 ± 0.58 ^d^
VSL (μm/s)	23.22 ± 0.34 ^b^	23.58 ± 0.57 ^b^	25.75 ± 0.98 ^a^	25.85 ± 0.70 ^a^	21.46 ± 0.27 ^c^
VCL (μm/s)	49.81 ± 0.22 ^b^	52.16 ± 0.65 ^b^	55.74 ± 0.89 ^a^	52.70 ± 1.34 ^ab^	47.55 ± 0.36 ^c^
VAP (μm/s)	36.98 ± 1.12 ^ab^	36.12 ± 1.02 ^ab^	37.75 ± 1.06 ^a^	37.66 ± 1.05 ^a^	33.98 ± 0.26 ^c^
ALH (μm)	15.66 ± 1.69 ^b^	15.38 ± 0.84 ^b^	15.98 ± 1.49 ^a^	15.96 ± 0.63 ^a^	14.90 ± 0.43 ^b^
WOB (%)	84.67 ± 4.90 ^a^	89.58 ± 1.08 ^a^	86.25 ± 4.05 ^a^	90.50 ± 1.08 ^a^	85.67 ± 2.12 ^b^
BCF (Hz)	0.76 ± 0.01 ^ab^	0.76 ± 0.01 ^ab^	0.78 ± 0.01 ^a^	0.77 ± 0.01 ^ab^	0.74 ± 0.00 ^c^
LIN (%)	43.92 ± 0.68 ^b^	44.58 ± 0.69 ^a^	45.92 ± 0.56 ^a^	45.83 ± 0.76 ^a^	42.58 ± 0.43 ^b^
MAD (°)	160.45 ± 23.30 ^bc^	202.37 ± 19.35 ^ab^	251.10 ± 24.24 ^a^	195.45 ± 11.37 ^ab^	145.89 ± 10.79 ^c^
STR (%)	59.58 ± 1.42 ^b^	63.83 ± 1.81 ^a^	64.17 ± 0.63 ^a^	63.58 ± 1.73 ^a^	56.50 ± 0.50 ^c^

Values are expressed as mean ± SEM (*n* = 5). Different superscript letters within the same row indicate significant differences (*p* < 0.05).

**Table 3:** Effects of different concentrations of MG on the thermo-resistance of boar sperm after thawing.

Group	1 h	2 h	3 h	4 h
Total motility				
0 μM	60.57 ± 0.64 ^c^	56.52 ± 0.43 ^c^	51.48 ± 0.77 ^c^	47.46 ± 0.62 ^c^
10 μM	66.70 ± 0.61 ^b^	65.47 ± 0.97 ^b^	61.17 ± 0.92 ^b^	56.01 ± 1.34 ^b^
20 μM	69.82 ± 0.23 ^a^	67.80 ± 1.39 ^a^	63.98 ± 0.28 ^a^	60.81 ± 0.42 ^a^
30 μM	67.80 ± 0.31 ^b^	58.88 ± 0.40 ^c^	56.61 ± 0.05 ^c^	51.75 ± 0.45 ^c^
50 μM	63.04 ± 0.19 ^c^	58.34 ± 0.07 ^c^	54.64 ± 0.51 ^c^	50.85 ± 0.30 ^c^
Progressive motility				
0 μM	45.23 ± 0.50 ^c^	42.01 ± 0.41 ^c^	38.58 ± 0.60 ^c^	35.95 ± 0.47 ^c^
10 μM	51.20 ± 0.55 ^b^	49.10 ± 0.82 ^b^	45.80 ± 0.75 ^b^	41.25 ± 0.96 ^b^
20 μM	54.80 ± 0.20 ^a^	52.65 ± 1.12 ^a^	48.25 ± 0.23 ^a^	44.05 ± 0.31 ^a^
30 μM	52.37 ± 0.28 ^b^	44.51 ± 0.35 ^c^	42.25 ± 0.03 ^c^	38.52 ± 0.39 ^c^
50 μM	48.10 ± 0.15 ^c^	44.05 ± 0.06 ^c^	40.20 ± 0.38 ^c^	36.15 ± 0.25 ^c^

Values are expressed as mean ± SEM (*n* = 5). Different superscript letters within the same column indicate significant differences (*p* < 0.05).

**Table 4:** Effects of MG supplementation in the freezing extender on in vitro fertilization (IVF) cleavage rates of boar sperm after thawing.

Groups	No. of Oocytes	No. of Cleaved	Cleavage Rate %
0 μM	220	113	51.36 ± 1.31 ^b^
20 μM	269	162	60.22 ± 1.27 ^a^
50 μM	285	130	45.61 ± 1.36 ^b^

Values are expressed as mean ± SEM (*n* = 5). Different superscript letters within the same column indicate significant differences (*p* < 0.05).

## Data Availability

The data presented in this study are available in the article. Further inquiries can be directed to the corresponding authors.

## References

[1] Deng S., Yang L., Gao L., Ning C., Wang S., Zhang W. (2025). The effect of combined cryoprotectants on the cryotolerance of boar sperm. Anim. Biosci..

[2] Bolarin A., Berndtson J., Tejerina F., Cobos S., Pomarino C., D’Alessio F., Blackburn H., Kaeoket K. (2024). Boar semen cryopreservation: State of the art, and international trade vision. Anim. Reprod. Sci..

[3] Pezo F., Romero F., Zambrano F., Sanchez R.S. (2019). Preservation of boar semen: An update. Reprod. Domest. Anim..

[4] Waberski D., Riesenbeck A., Schulze M., Weitze K.F., Johnson L. (2019). Application of preserved boar semen for artificial insemination: Past, present and future challenges. Theriogenology.

[5] Yanez-Ortiz I., Catalan J., Rodriguez-Gil J.E., Miro J., Yeste M. (2022). Advances in sperm cryopreservation in farm animals: Cattle, horse, pig and sheep. Anim. Reprod. Sci..

[6] Zhang B., Wang Y., Wu C., Qiu S., Chen X., Cai B., Xie H. (2021). Freeze-thawing impairs the motility, plasma membrane integrity and mitochondria function of boar spermatozoa through generating excessive ROS. BMC Vet. Res..

[7] O’Flaherty C., Scarlata E. (2022). OXIDATIVE STRESS AND REPRODUCTIVE FUNCTION: The protection of mammalian spermatozoa against oxidative stress. Reproduction.

[8] Zhang W., Min L., Li Y., Lang Y., Hoque S.A.M., Adetunji A.O., Zhu Z. (2022). Beneficial Effect of Proline Supplementation on Goat Spermatozoa Quality during Cryopreservation. Animals.

[9] Hungerford A., Bakos H.W., Aitken R.J. (2023). Sperm cryopreservation: Current status and future developments. Reprod. Fertil. Dev..

[10] Chianese R., Pierantoni R. (2021). Mitochondrial Reactive Oxygen Species (ROS) Production Alters Sperm Quality. Antioxidants.

[11] Zhang L., Sun Y., Jiang C., Sohail T., Sun X., Wang J., Li Y. (2024). Damage to Mitochondria During the Cryopreservation, Causing ROS Leakage, Leading to Oxidative Stress and Decreased Quality of Ram Sperm. Reprod. Domest. Anim..

[12] Andrabi S.W., Ara A., Saharan A., Jaffar M., Gugnani N., Esteves S.C. (2024). Sperm DNA Fragmentation: Causes, evaluation and management in male infertility. JBRA Assist. Reprod..

[13] Feng Q., Yang Y., Zhang B., Shi W., Fang Y., Xu C., Deng Z., Feng W., Shi D. (2025). The Effects of Limonin, Myo-Inositol, and L-Proline on the Cryopreservation of Debao Boar Semen. Animals.

[14] Kujoana T.C., Sehlabela L.D., Mabelebele M., Sebola N.A. (2024). The potential significance of antioxidants in livestock reproduction: Sperm viability and cryopreservation. Anim. Reprod. Sci..

[15] Sanchez-Rubio F., Soria-Meneses P.J., Jurado-Campos A., Bartolome-Garcia J., Gomez-Rubio V., Soler A.J., Arroyo-Jimenez M.M., Santander-Ortega M.J., Plaza-Oliver M., Lozano M.V. (2020). Nanotechnology in reproduction: Vitamin E nanoemulsions for reducing oxidative stress in sperm cells. Free Radic. Biol. Med..

[16] Hungerford A.J., Bakos H.W., Aitken R.J. (2024). Addition of Vitamin C Mitigates the Loss of Antioxidant Capacity, Vitality and DNA Integrity in Cryopreserved Human Semen Samples. Antioxidants.

[17] Kaeoket K., Chanapiwat P. (2023). The Beneficial Effect of Resveratrol on the Quality of Frozen-Thawed Boar Sperm. Animals.

[18] Bang S., Tanga B.M., Fang X., Seong G., Saadeldin I.M., Qamar A.Y., Lee S., Kim K., Park Y., Nabeel A.H.T. (2022). Cryopreservation of Pig Semen Using a Quercetin-Supplemented Freezing Extender. Life.

[19] Wang S., Wang Q., Min L., Cao H., Adetunji A.O., Zhou K., Zhu Z. (2025). Pyrroloquinoline Quinone Improved Boar Sperm Quality via Maintaining Mitochondrial Function During Cryopreservation. Antioxidants.

[20] Hai E., Li B., Zhang J., Zhang J. (2024). Sperm freezing damage: The role of regulated cell death. Cell Death Discov..

[21] Ozimic S., Ban-Frangez H., Stimpfel M. (2023). Sperm Cryopreservation Today: Approaches, Efficiency, and Pitfalls. Curr. Issues Mol. Biol..

[22] Berean D.I., Bogdan L.M., Cimpean R. (2024). Advancements in Understanding and Enhancing Antioxidant-Mediated Sperm Cryopreservation in Small Ruminants: Challenges and Perspectives. Antioxidants.

[23] Liang H., Huang Q., Zou L., Wei P., Lu J., Zhang Y. (2023). Methyl gallate: Review of pharmacological activity. Pharmacol. Res..

[24] Neo S., Siew Y., Yew H., He Y., Poh K., Tsai Y., Ng S., Tan W., Chong T., Lim C.S.E. (2023). Effects of Leea indica leaf extracts and its phytoconstituents on natural killer cell-mediated cytotoxicity in human ovarian cancer. BMC Complement. Med. Ther..

[25] Zhang X., Wu Z., Weng P. (2014). Antioxidant and hepatoprotective effect of (-)-epigallocatechin 3-O-(3-O-methyl) gallate (EGCG3′′Me) from Chinese oolong tea. J. Agric. Food. Chem..

[26] Li Y., Shen B., Lv C., Zhu X., Naren Q., Xu D., Chen H., Wu F. (2023). Methyl gallate prevents oxidative stress induced apoptosis and ECM degradation in chondrocytes via restoring Sirt3 mediated autophagy and ameliorates osteoarthritis progression. Int. Immunopharmacol..

[27] Rosas E.C., Correa L.B., Padua T.D.A., Costa T.E.M.M., Mazzei J.L., Heringer A.P., Bizarro C.A., Kaplan M.A.C., Figueiredo M.R., Henriques M.G. (2015). Anti-inflammatory effect of Schinus terebinthifolius Raddi hydroalcoholic extract on neutrophil migration in zymosan-induced arthritis. J. Ethnopharmacol..

[28] Ferreira N.S., Cascaes M.M., Da Silva Santos L., de Oliveira M.S., Das Gracas Bichara Zoghbi M., Araujo I.S., Uetanabaro A.P.T., de Aguiar Andrade E.H., Guilhon G.M.S.P. (2022). Flavanone Glycosides, Triterpenes, Volatile Compounds and Antimicrobial Activity of Miconia minutiflora (Bonpl.) DC. (Melastomataceae). Molecules.

[29] Wang C., Safwan S., Cheng M., Liao T., Cheng L., Chen T., Kuo Y., Lin Y., Lee C. (2020). Protective Evaluation of Compounds Extracted from Root of Rhodiola rosea L. against Methylglyoxal-Induced Toxicity in a Neuronal Cell Line. Molecules.

[30] Whang W.K., Park H.S., Ham I.H., Oh M., Namkoong H., Kim H.K., Hwang D.W., Hur S.Y., Kim T.E., Park Y.G. (2005). Methyl gallate and chemicals structurally related to methyl gallate protect human umbilical vein endothelial cells from oxidative stress. Exp. Mol. Med..

[31] Park D.J., Jung H.J., Park C.H., Yokozawa T., Jeong J. (2019). Root Bark of Paeonia suffruticosa Extract and Its Component Methyl Gallate Possess Peroxynitrite Scavenging Activity and Anti-inflammatory Properties through NF-kappaB Inhibition in LPS-treated Mice. Molecules.

[32] Ryu B., Kim K. (2022). Antioxidant activity and protective effect of methyl gallate against t-BHP induced oxidative stress through inhibiting ROS production. Food Sci. Biotechnol..

[33] Ribas-Maynou J., Mateo-Otero Y., Delgado-Bermudez A., Bucci D., Tamanini C., Yeste M., Barranco I. (2021). Role of exogenous antioxidants on the performance and function of pig sperm after preservation in liquid and frozen states: A systematic review. Theriogenology.

[34] Sui H., Wang X., Hu K., Zuo X., Li H., Diao Z., Feng J., Zhang Y., Cao Z. (2025). Effects of Mogroside V on Quality and Antioxidant Activity of Boar Frozen-Thawed Sperm. Antioxidants.

[35] Almubarak A., Lee S., Yu I., Jeon Y. (2024). Effects of Nobiletin supplementation on the freezing diluent on porcine sperm cryo-survival and subsequent in vitro embryo development. Theriogenology.

[36] Mili B., Chutia T., Buragohain L., Palanisammi A., Kumaresan A. (2025). Liquid storage of boar semen: Current approaches to reducing sperm damage using antioxidants and nanotechnology. Anim. Reprod. Sci..

[37] Galarza D.A., Jaramillo J., Amon N., Campoverde B., Aguirre B., Taboada J., Samaniego X., Duma M. (2024). Effect of resveratrol supplementation in conventional slow and ultra-rapid freezing media on the quality and fertility of bull sperm. Anim. Reprod. Sci..

[38] Yue S., Wang S., Liu X., Bian X., Ding C., Wu T., Li D., Zhou J. (2023). Ameliorative effect of silymarin on the quality of frozen-thawed boar spermatozoa. Reprod. Domest. Anim..

[39] Ahmed H., Jahan S., Riaz M., Khan B.T., Ijaz M.U. (2020). Epigallocatechin-3-gallate (EGCG) addition as an antioxidant in a cryo-diluent media improves microscopic parameters, and fertility potential, and alleviates oxidative stress parameters of buffalo spermatozoa. Cryobiology.

[40] Bisht S., Dada R. (2017). Oxidative stress: Major executioner in disease pathology, role in sperm DNA damage and preventive strategies. Front. Biosci. (Schol. Ed.).

[41] Zhou D., Wang X., Li R., Wang Y., Chao Y., Liu Z., Huang Z., Nie H., Zhu W., Tan Y. (2021). Improving native human sperm freezing protection by using a modified vitrification method. Asian J. Androl..

[42] Garcia-Vazquez F.A., Gadea J., Matas C., Holt W.V. (2016). Importance of sperm morphology during sperm transport and fertilization in mammals. Asian J. Androl..

[43] Liao Q., Huang B., Zhang S., Chen J., Chen G., Li K., Ai J. (2020). Influence of Different Quality Sperm on Early Embryo Morphokinetic Parameters and Cleavage Patterns: A Retrospective Time-lapse Study. Curr. Med. Sci..

[44] Li Y., Kalo D., Zeron Y., Roth Z. (2016). Progressive motility—A potential predictive parameter for semen fertilization capacity in bovines. Zygote.

[45] Zaloga G.P. (2021). Narrative Review of n-3 Polyunsaturated Fatty Acid Supplementation upon Immune Functions, Resolution Molecules and Lipid Peroxidation. Nutrients.

[46] Lu Y., Pan Y., Sheng N., Zhao A.Z., Dai J. (2016). Perfluorooctanoic acid exposure alters polyunsaturated fatty acid composition, induces oxidative stress and activates the AKT/AMPK pathway in mouse epididymis. Chemosphere.

[47] Guo H., Wang J., Sun L., Jin X., Shi X., Lin J., Yue S., Zhou J. (2021). Effects of astaxanthin on plasma membrane function and fertility of boar sperm during cryopreservation. Theriogenology.

[48] Mohideen K., Chandrasekar K., Ramsridhar S., Rajkumar C., Ghosh S., Dhungel S. (2023). Assessment of Oxidative Stress by the Estimation of Lipid Peroxidation Marker Malondialdehyde (MDA) in Patients with Chronic Periodontitis: A Systematic Review and Meta-Analysis. Int. J. Dent..

[49] Li L., Feng T., Wu R., Zhang Y., Wang N., Wu M., Pang Y., Yang S., Yang A., Zhang D. (2023). The role of total antioxidant capacity and malondialdehyde of seminal plasma in the association between air pollution and sperm quality. Environ. Pollut..

[50] Alyethodi R.R., Sirohi A.S., Karthik S., Tyagi S., Perumal P., Singh U., Sharma A., Kundu A. (2021). Role of seminal MDA, ROS, and antioxidants in cryopreservation and their kinetics under the influence of ejaculatory abstinence in bovine semen. Cryobiology.

[51] Barati E., Nikzad H., Karimian M. (2020). Oxidative stress and male infertility: Current knowledge of pathophysiology and role of antioxidant therapy in disease management. Cell. Mol. Life Sci..

[52] Liu D., Qu J., Zhan Y., Wu X., Qiao H., Zhang Y., Li N., Li B. (2025). Evaluation of intracellularly targeted engineered antioxidant fusion proteins SOD-LCA2 and Prx-LCA2 as promising therapeutic combinations for alleviating and restoring pulmonary oxidative damage. Int. J. Biol. Macromol..

[53] Hsieh T., Liu T., Chia Y., Chern C., Lu F., Chuang M., Mau S., Chen S., Syu Y., Chen C. (2004). Protective effect of methyl gallate from Toona sinensis (Meliaceae) against hydrogen peroxide-induced oxidative stress and DNA damage in MDCK cells. Food. Chem. Toxicol..

[54] Collin F. (2019). Chemical Basis of Reactive Oxygen Species Reactivity and Involvement in Neurodegenerative Diseases. Int. J. Mol. Sci..

[55] Agarwal A., Sharma R.K., Sharma R., Assidi M., Abuzenadah A.M., Alshahrani S., Durairajanayagam D., Sabanegh E. (2014). Characterizing semen parameters and their association with reactive oxygen species in infertile men. Reprod. Biol. Endocrinol..

[56] Luo S., Schatten H., Sun Q. (2013). Sperm mitochondria in reproduction: Good or bad and where do they go?. J. Genet. Genom..

[57] Koppers A.J., De Iuliis G.N., Finnie J.M., Mclaughlin E.A., Aitken R.J. (2008). Significance of mitochondrial reactive oxygen species in the generation of oxidative stress in spermatozoa. J. Clin. Endocrinol. Metab..

[58] Aitken R.J., Baker M.A. (2006). Oxidative stress, sperm survival and fertility control. Mol. Cell. Endocrinol..

[59] Said T.M., Gaglani A., Agarwal A. (2010). Implication of apoptosis in sperm cryoinjury. Reprod. Biomed. Online.

[60] Tang D., Kang R., Berghe T.V., Vandenabeele P., Kroemer G. (2019). The molecular machinery of regulated cell death. Cell Res..

[61] Renault T.T., Dejean L.M., Manon S. (2017). A brewing understanding of the regulation of Bax function by Bcl-xL and Bcl-2. Mech. Ageing. Dev..

[62] Li C., Oh H.J., Liu H., Kim M.K. (2023). Schisandrin B protects boar spermatozoa against oxidative damage and increases their fertilization ability during in vitro storage. Theriogenology.

[63] Babaei F., Khoshsokhan Muzaffar M., Jannatifar R. (2024). The association of sperm BAX and BCL-2 gene expression with reproductive outcome in Oligoasthenoteratozoospermia cases undergoing intracytoplasmic sperm injection: A case-control study. Int. J. Reprod. Biomed..

[64] Sapanidou V., Tsantarliotou M.P., Feidantsis K., Tzekaki E.E., Kourousekos G., Lavrentiadou S.N. (2025). Supplementing Freezing Medium with Crocin Exerts a Protective Effect on Bovine Spermatozoa Through the Modulation of a Heat Shock-Mediated Apoptotic Pathway. Molecules.

[65] Huang C., Chang Y., Wei P., Hung C., Wang W. (2021). Methyl gallate, gallic acid-derived compound, inhibit cell proliferation through increasing ROS production and apoptosis in hepatocellular carcinoma cells. PLoS ONE.

[66] Xue S., Xu B., Yan X., Zhang J., Su R. (2025). Sperm Membrane Stability: In-Depth Analysis from Structural Basis to Functional Regulation. Vet. Sci..

[67] Kalthur G., Raj S., Thiyagarajan A., Kumar S., Kumar P., Adiga S.K. (2011). Vitamin E supplementation in semen-freezing medium improves the motility and protects sperm from freeze-thaw-induced DNA damage. Fertil. Steril..

